# Supplementary material for: An investigation on the energy absorption characteristics of a multi-cell hexagonal tube under axial crushing loads
Source: PLoS One. 2020 Jun 8;15(6):e0233708. doi: 10.1371/journal.pone.0233708 (PMC7279609; doi:10.1371/journal.pone.0233708)
Supplement: S1 Table — (PDF) [file pone.0233708.s001.pdf]

S1 Table. Crashworthiness characteristics of the multi-cell hexagonal tube

No.	C (mm)	t_1 (mm)	t_2 mm)	M (kg)	EA (kJ)	SEA (kJ/kg)	F (kN）
1	45	2	2	5.21	62.82	12.1	260.83
2	45	2	2.5	5.63	68.89	12.2	306.76
3	45	2	3	6.06	74.69	12.3	340.92
4	45	2.5	2	6.08	88.36	14.5	322.02
5	45	2.5	2.5	6.51	93.70	14. 4	359.65
6	45	2.5	3	6.94	101.60	14.6	394.06
7	45	3	2	6.96	114.37	16.4	390.90
8	45	3	2.5	7.38	122.35	16.6	423.58
9	45	3	3	7.81	129.32	16.5	454.00
10	50	2	2	5.35	63.41	11.8	269.18
11	50	2	2.5	5.72	68.22	11.9	300.85
12	50	2	3	6.09	73.69	12.1	332.19
13	50	2.5	2	6.32	87.52	13.8	333.61
14	50	2.5	2.5	6.69	95.42	14.2	362.00
15	50	2.5	3	7.06	100.32	14.2	391.23
16	50	3	2	7.30	116.25	15.9	404.26
17	50	3	2.5	7.66	121.55	15.8	429.75
18	50	3	3	8.03	130.94	16.2	459.50
19	55	2	2	5.50	62.99	11.4	273.63
20	55	2	2.5	5.81	67.91	11.7	298.37
21	55	2	3	6.11	73.06	11.9	326.58
22	55	2.5	2	6.57	88.81	13.5	346.36

23	55	2.5	2.5	6.88	93.05	13.5	369.24
24	55	2.5	3	7.18	98.63	13.7	395.77
25	55	3	2	7.64	117.97	15.4	422.78
26	55	3	2.5	7.95	123.80	15.6	446.22
27	55	3	3	8.25	130.27	15.8	472.39
28	60	2	2	5.65	63.21	11.2	280.49
29	60	2	2.5	5.89	67.57	11.5	303.15
30	60	2	3	6.14	72.36	11.8	321.25
31	60	2.5	2	6.81	87.43	12.8	358.11
32	60	2.5	2.5	7.06	93.11	13.1	377.87
33	60	2.5	3	7.31	99.22	13.6	400.27
34	60	3	2	7.98	118.39	14.8	444.81
35	60	3	2.5	8.23	125.01	15.2	464.93
36	60	3	3	8.47	130.81	15.4	487.01
37	65	2	2	5.79	63.06	10.9	280.68
38	65	2	2.5	5.98	66.26	11.1	296.51
39	65	2	3	6.17	68.96	11.2	312.14
40	65	2.5	2	7.06	87.30	12.4	362.82
41	65	2.5	2.5	7.24	91.76	12.7	381.89
42	65	2.5	3	7.43	96.94	13.0	400.62
43	65	3	2	8.32	118.19	14.2	460.98
44	65	3	2.5	8.51	122.05	14.3	478.58
45	65	3	3	8.69	127.46	14.7	498.90
