# Supplementary material for: An investigation on the energy absorption characteristics of a multi-cell hexagonal tube under axial crushing loads
Source: PLoS One. 2020 Jun 8;15(6):e0233708. doi: 10.1371/journal.pone.0233708 (PMC7279609; doi:10.1371/journal.pone.0233708)
Supplement: Attachment — Submitted filename: Response to Reviewers.docx [file pone.0233708.s003.docx]

Dear Editors and Reviewers:

Thank you for your letter and for the reviewers’ comments concerning our manuscript entitled " An investigation on the energy absorption characteristics of a multi-cell hexagonal tube under axial crushing loads" (ID: PONE-D-19-30455). Your comments and those of the reviewers were highly insightful and enabled us to greatly improve the quality of our manuscript. We have studied comments carefully and have made correction which we hope meet with approval. All the revisions in the manuscript and the supporting information have been highlighted in red color. In the following pages are our point-by-point responses to each of the comments of the reviewers.

We greatly appreciate your time and efforts to improve our manuscript for publication.

Sincerely,

MingKai Yue

**Response to the Editor**

Thank you for your comments. Considering your suggestion, we have added the stress contours in Fig. 3.

Fig. 3 The stress contours collapse of typical angle elements

**Response to the Journal Requirements**

***Comment1:*** *Please ensure that your manuscript meets PLOS ONE's style requirements, including those for file naming.*

**Response:** Thank you for your comments. We have revised the style for our manuscript based on the requirements.

***Comment2:*** *We note that Figure 8b in your submission contain copyrighted images.*

**Response:** Figure 8b consists two pictures, the first is quoted from the existing literature and the second is the result of our own simulation. For copyright reasons, we removed the first picture and only kept our own results.

***Comment3:*** *We note that you have stated that you will provide repository information for your data at acceptance. Should your manuscript be accepted for publication, we will hold it until you provide the relevant accession numbers or DOIs necessary to access your data. If you wish to make changes to your Data Availability statement, please describe these changes in your cover letter and we will update your Data Availability statement to reflect the information you provide.*

**Response:** Thank you very much for your reminder. There no changes in this part at present.

***Comment4:*** *PLOS requires an ORCID iD for the corresponding author in Editorial Manager on papers submitted after December 6th, 2016. Please ensure that you have an ORCID iD and that it is validated in Editorial Manager.*

**Response:** Thank you very much for your reminder. We have applied for a ORCID iD (0000-0002-6833-6462). But when click on the Fetch/Validate link next to the ORCID field, windows always prompt "Redirect URI doesn't match your registered redirect URIs". Could you give us some suggestions to solve it?

***Comment5:*** *Please include captions for your Supporting Information files at the end of your manuscript, and update any in-text citations to match accordingly.*

**Response:** Thank you for your comments. We have added Supporting Information files and update the citations in manuscript.

**Response to the reviewer**

***Comment1:*** *The paper is very interesting and the topic of the paper falls within the scope of the journal. Overall, the paper is well organized and written.*

**Response:** Thank you for your constructive comments concerning our manuscript.

***Comment2:*** *The experimental and numerical analysis are too superficial, please add some analysis on the thin-walled structures under different loading angles.*

**Response:** Thanks for your suggestion. It is really true as reviewer suggested that the loading conditions are too superficial. The energy absorber proposed in this paper was designed to improve the crashworthiness of railway vehicles. The train buffer is always subjected to the loading in the direction of moving under the constraint of railway. According to statistics, almost all train collisions involve frontal crash. According to the requirements of standards for train crashworthiness(BS EN 15227-2008), the structure shall meet the frontal impact condition with the speed of 10m/s. So, this paper only considered the crashworthiness under frontal impact condition to meeting the requirements of standard. Oblique loading condition involves complex multi-directional instability, which will be our future work direction. We have added the following explanation in manuscript:

This paper aims to investigate the energy absorption of a new multi-cell tube design which was designed to improve the crashworthiness of railway vehicles. The train buffer is always subjected to the loading in the direction of moving under the constraint of railway. According to statistics, almost all train collisions involve frontal crash.

According to the requirements of standards for train crashworthiness(BS EN 15227-2008) [43], the structure shall meet the frontal impact condition with the speed of 10m/s. So, this paper considered the crashworthiness under frontal impact condition to meeting the requirements of standard.

[43] Technical Committee CEN/TC 256 Railway Applications, BS EN 15227-2008 Railway Applications: Crashworthiness Requirements for Railway Vehicle Bodies, British Standard Institution, London, 2008.

***Comment3:*** *Some very important pioneering works in the subject area are missing and should be well discussed in Introduction, especially some research on the thin-walled structures under crashworthiness.*

**Response:** Considering the Reviewer’s suggestion, we have revised the Introduction and literature review. The application of thin-walled structures in the energy absorption system has been added in the manuscript. The pioneering works have also been introduced through the existing literature. The added literatures are as follows:

[5] Andrews K R F, England G L, Ghani E . Classification of the axial collapse of cylindrical tubes under quasi-static loading[J]. International Journal of Mechanical Sciences, 1983, 25(9-10):687-696.

[6] Wlodzimierz, Abramowicz, and, et al. Dynamic axial crushing of square tubes[J]. International Journal of Impact Engineering, 1984.

[7] Abramowicz W, Jones N. Dynamic axial crushing of circular tubes[J]. International Journal of Impact Engineering, 1984, 2(3):263-281.

[8] X. Zhang, G. Cheng, A comparative study of energy absorption characteristics of foam-filled and multi-cell square columns, Int. J. Impact Eng. 34 (11) (2007)

1739–1752.

[9] Gao G, Dong H, Tian H. Collision performance of square tubes with diaphragms[J]. Thin-Walled Structures, 2014, 80:167-177.

[14] Guangyong, S. On crashing behaviors of aluminium/CFRP tubes subjected to axial and oblique loading: An experimental study[J]. Composites Part B: Engineering, 2018.

[15] Liu Q, Xu X, Ma J. Lateral crushing and bending responses of CFRP square tube filled with aluminum honeycomb [J]. Composites, 2017, 118B(JUN.):104-115.

[18] Nia A, Kazemi M. Experimental study of ballistic resistance of sandwich targets with aluminum face-sheet and graded foam core[J]. Journal of Sandwich Structures & Materials, 2018:109963621875766.

[28] Qiu N , Gao Y , Fang J , et al. Theoretical prediction and optimization of multi-cell hexagonal tubes under axial crashing[J]. Thin-Walled Structures, 2016, 102:111-121.

[32] Qiu N , Gao Y , Fang J , et al. Topological design of multi-cell hexagonal tubes under axial and lateral loading cases using a modified particle swarm algorithm[J]. Applied Mathematical Modelling, 2018, 53(JAN.):567-583.

[42] Pang T, Zheng G, Fang J, Energy absorption mechanism of axially-varying thickness (AVT) multicell thin-walled structures under out-of-plane loading. Engineering Structures 2019;196:112-122.

***Comment4:*** *Please expand figure captions so figures are almost self explanatory.*

**Response:** We have improved our paper according to the reviewer's comment. The **Fig.1**, **Fig.2**, **Fig.3**, **Fig.6 Fig.7**, **Fig.8**, **Fig.10**, **Fig.11 and Fig.12** have been revised.

**Once again, thank you very much for your comments and suggestions.**
